# Transglutaminase-Induced Polymerization of Pea and Chickpea Protein to Enhance Functionality

**DOI:** 10.3390/gels10010011

**Published:** 2023-12-22

**Authors:** Brigitta P. Yaputri, Samira Feyzi, Baraem P. Ismail

**Affiliations:** Food Science and Nutrition Department, University of Minnesota, 1334 Eckles Ave., Saint Paul, MN 55108, USA; yaput001@umn.edu (B.P.Y.); sfeyzi@umn.edu (S.F.)

**Keywords:** pea protein isolate, chickpea protein isolate, salt extraction coupled with ultrafiltration, transglutaminase-induced polymerization, functionality

## Abstract

Pulse proteins, such as pea and chickpea proteins, have inferior functionality, specifically gelation, compared to soy protein, hindering their applications in different food products, such as meat analogs. To close the functionality gap, protein polymerization via targeted modification can be pursued. Accordingly, transglutaminase-induced polymerization was evaluated in pea protein isolate (PPI) and chickpea protein isolate (ChPI) to improve their functionality. The PPI and ChPI were produced following a scaled-up salt extraction coupled with ultrafiltration (SE-UF) process. Transglutaminase (TGase)-modified PPI and ChPI were evaluated in comparison to unmodified counterparts and to commercial protein ingredients. Protein denaturation and polymerization were observed in the TG PPI and TG ChPI. In addition, the TGase modification led to the formation of intermolecular β-sheet and β-turn structures that contributed to an increase in high-molecular-weight polymers, which, in turn, significantly improved the gel strength. The TG ChPI had a significantly higher gel strength but a lower emulsification capacity than the TG PPI. These results demonstrated the impact of the inherent differences in the protein fractions on the functional behavior among species. For the first time, the functional behavior of the PPI and ChPI, produced on a pilot scale under mild processing conditions, was comprehensively evaluated as impacted by the TGase-induced structural changes.

## 1. Introduction

The market value of plant protein ingredients is increasing as the demand for plant-based food and beverages continues to grow. The global market size for plant-based products reached USD 10.24 billion in 2022 and is expected to grow at a CAGR of 11.8%, reaching USD 22.3 billion by 2029 [1]. In the US alone, the plant-based market was valued at USD 8 billion in 2022, a 6.6% increase from 2021 [2]. Meat analogs, which are produced using various plant protein ingredients, are among the top three categories in the plant-based market [3,4,5].

Soy protein and wheat gluten are most commonly used in meat analogs, as they contain high-molecular-weight proteins with an abundance of sulfhydryl groups. Upon extrusion, these high-molecular-weight proteins form disulfide bonds and other intermolecular linkages, resulting in strong protein networks of a meat-like fibrous structure [4,6,7]. However, soy and wheat gluten are listed among the “Big Nine” allergens, and soy in the US is mostly genetically modified (GMO), raising skepticism and concerns among consumers [8,9]. In addition, wheat gluten is a concern for individuals with celiac disease or gluten sensitivity, who represent 1% and 6% of the US population, respectively [10,11]. As a result, consumers are seeking alternative plant proteins in their diets, driving the food industry to consider other protein sources that can provide similar functional and textural properties to soy protein and wheat gluten. 

Pea protein and chickpea protein obtained from yellow field peas (*Pisum sativum* L.) and chickpea (*Cicer arietinum* L.), respectively, have a similar protein profile and quality compared to soy protein [12,13,14]. Thus, the interest in pea and chickpea protein ingredients is on the rise as potential substitutes for soy protein in food and beverage applications, including meat analogs [15,16,17]. Compared to soy protein, however, pea and chickpea protein tend to have inferior functionality, especially gelation, which, in turn, contributes to inferior texturization properties [14,18,19].

Commercial pea and chickpea proteins, commonly produced using alkaline extraction coupled by isoelectric precipitation (AE-IEP), have relatively poor gelation properties, mainly attributed to harsh extraction conditions that result in denatured and extensively polymerized proteins [12,14,20,21]. In previous studies, salt extraction coupled with ultrafiltration (SE-UF) was found to be an efficient and scalable protein extraction method that preserved the structure of the pea and chickpea proteins [12,14]. Pea and chickpea protein isolates (PPIs and ChPIs) produced following SE-UF was relatively less denatured and had superior functional properties compared to commercial pea and chickpea protein ingredients. However, both isolates remained inferior to the commercial soy protein isolate (cSPI) in gel strength [12,14]. The structural modification of PPI and ChPI may be required to further enhance their gel strength.

Gel strength is an assessment of the cohesiveness of a protein network that is stabilized by noncovalent interactions and disulfide bonds [7,22]. The formation of high-molecular-weight (HMW) soluble aggregates may contribute to an enhanced gel strength [23]. Protein crosslinking to form soluble aggregates can be achieved via different approaches, including cold atmospheric plasma, high-pressure processing, and thermal processing [24,25,26]. However, cold atmospheric plasma is not yet a scalable approach for protein ingredients [24], and high-pressure and thermal processing might result in the formation of excess insoluble aggregates that can negatively impact the functionality and nutritional quality [27,28,29]. Enzymatic modification using transglutaminase has also been recently explored to induce protein crosslinking [30,31,32]. Transglutaminase (TGase) catalyzes the acyl transfer between the carboxamide group of a glutamine residue and the amine of a lysine residue, forming inter- and intramolecular covalent crosslinks that result in the formation of HMW soluble aggregates [33,34]. TGase is considered as a GRAS and has been used in meat, seafood, tofu, dairy, and plant-based products to induce protein polymerization, which enhances the functional and textural properties [35,36,37,38,39].

Although a few studies have shown that pea and chickpea protein can be suitable substrates for TGase modification due to their relatively high lysine content [20,32,40,41,42], comprehensive research, including the selection of parameters for TGase treatment and their impact on the structure and function of the PPI and ChPI produced following scaled-up SE-UF, have not been reported. Therefore, the objective of this study was to evaluate the impact of the selected parameters of TGase-induced polymerization on the structural and functional properties of the PPI and ChPI produced following a pilot-scale SE-UF process in comparison to unmodified isolates and commercial protein ingredients.

## 2. Results and Discussion

### 2.1. Impact of the TG Treatment on the Protein Structural Characteristics 

#### 2.1.1. Protein Profile

**Figure 1:**
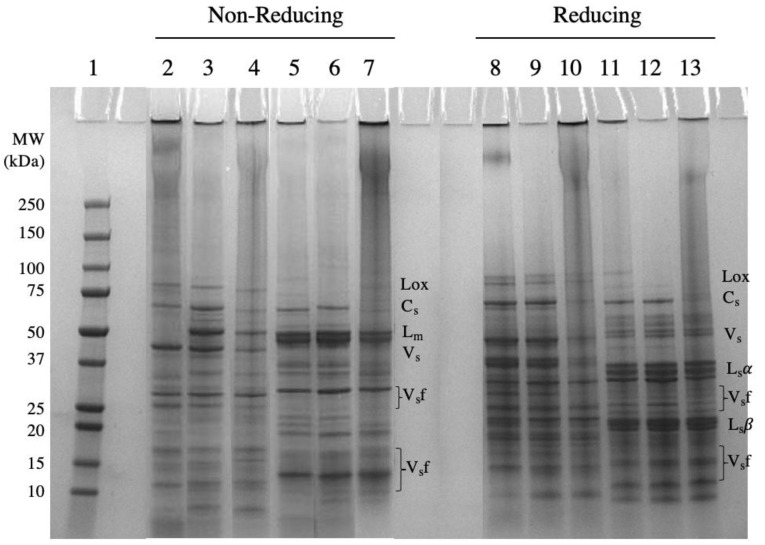
SDS-PAGE gel protein profile visualization of unmodified and TGase-modified pea and chickpea protein isolates (PPI, ChPI, TG PPI, and TG ChPI), as well as commercial PPI and ChPC (cPPI and cChPC) under nonreducing (lanes 2–7) and reducing (lanes 8–13) conditions. Lane 1: molecular weight (MW) marker; lanes 2, 8: cPPI reference; lanes 3, 9: PPI; lanes 4, 10: TG PPI; lanes 5, 11: cChPC reference; lanes 6, 12: ChPI; lanes 7, 13: TG ChPI. Lox: lipoxygenase; Cs: convicilin subunits; Lm: legumin monomer; Vs: vicilin subunits; Lsα: legumin acidic subunits, Lsβ: legumin basic subunits; Vsf: vicilin subunit fractions due to post-translational cleavages.

The protein profile of the TG PPI and TG ChPI was compared to the unmodified isolates and commercial samples under nonreducing and reducing conditions (Figure 1). The pea and chickpea protein samples had protein bands corresponding to convicilin (70 kDa), legumin monomer (60 kDa) and its acidic (40 kDa) and basic (20 kDa) subunits (seen under reducing conditions), and vicilin subunits (50 kDa, 30–35 kDa, and 13–19 kDa), similar to the protein profile reported by others [12,14,43,44]. Compared to both unmodified protein isolates (Figure 1, lanes 3 and 6), the cPPI showed intense smearing under nonreducing conditions in the upper region of its lane, with the absence of a legumin band at 60 kDa (Figure 1, lane 2), indicating polymerization involving mainly legumins [14]. Residual smearing and banding persisted in the cPPI and cChPC under reducing conditions (Figure 1, lanes 8 and 11), confirming the presence of protein polymerization driven by other covalent bonding, and not only disulfide linkages [45,46]. Such an extent of protein polymerization in commercial ingredients is often attributed to harsh extraction conditions, which have been reported to impair the functionality, specifically the solubility and gel strength [12,14,47]. In contrast, limited protein polymerization was noted in the PPI and ChPI produced by the SE-UF process (Figure 1, lanes 9 and 12), similar to the observations by Hansen et al. [12] and Yaputri et al. [14]. 

On the other hand, the TG PPI and TG ChPI demonstrated protein polymerization that persisted under reducing conditions (Figure 1, lanes 4, 7, 10, and 13), confirming covalent interactions induced by the TGase treatment. Intense smearing was accompanied by less-apparent bands corresponding to legumin monomers (60 kDa), convicilin (70 kDa), and vicilin subunits (50 kDa, 30–35 kDa) in both the TG PPI and TG ChPI (Figure 1, lanes 4 and 7). However, smaller-molecular-weight bands (<25 kDa) corresponding to small vicilin subunits (~20 kDa) did not appear to have taken part in the noted polymerization, as was observed by Djoullah et al. [40], Romano et al. [48], and Zhao et al. [49] for pea globulin fraction, white bean flour, and cPPI. In addition, the legumin acidic (α) subunit (~40 kDa) of the TG PPI, but not the TG ChPI, was less apparent compared to the basic subunit (~20 kDa) (Figure 1, lane 10), suggesting that the α-subunits of the PPI were more susceptible to TGase-induced crosslinking. This observation can potentially be attributed to their relatively high amount of glutamine and lysine residues [30,40,42]. The observed polymerization of the TGase-modified isolates would likely impact the other structural properties, which may consequently affect the functional properties.

#### 2.1.2. Protein Molecular Weight Distribution and Polymerization

**Figure 2:**
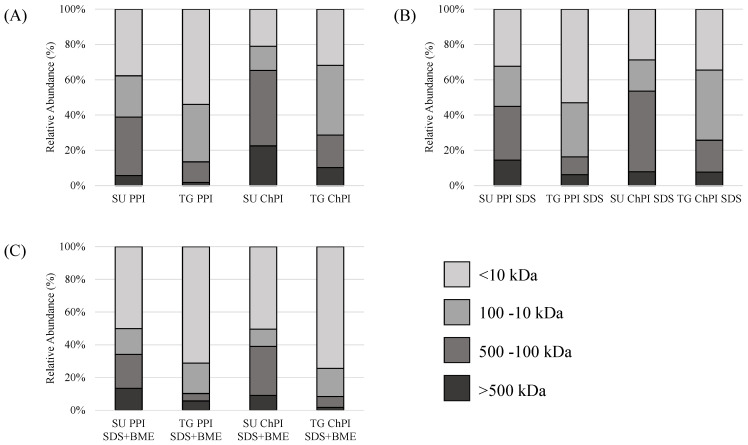
Percent relative abundance of major protein fractions in unmodified and TGase-modified pea and chickpea protein isolates (PPI, ChPI, TG PPI, and TG ChPI) as analyzed by SE-HPLC at a pH of 7.0 in (**A**) phosphate buffer, (**B**) 0.1% SDS phosphate buffer, and (**C**) 0.1% SDS and 2.5% βME phosphate buffer. The bar distribution represents means of *n* = 2.

The protein molecular weight distribution, and the relative abundance of soluble aggregates and major protein fractions, are shown in Table 1 and Figure 2. Fractions over 500 kDa were considered as soluble aggregates, since they were larger than the ~450 kDa hexameric form of legumin [23]. When dissolved in phosphate buffer, both the TG PPI and TG ChPI had a significantly lower relative abundance of soluble aggregates and functional proteins compared to the PPI and ChPI (Table 1 and Figure 2A). This observation confirmed that most functional proteins in the TGase-modified isolates were involved in the formation of large-molecular-weight aggregates that did not pass through the 0.45 µm filter. Compared to the PPI, the ChPI had a significantly higher relative abundance of soluble aggregates, attributed to its higher relative abundance of 11S legumin [14]. The relative abundance of legumin in both the TG PPI and TG ChPI was significantly lower than their unmodified counterparts (Table 1), confirming the involvement of legumin in the formation of large-molecular-weight polymers upon treatment with TG (Figure 1).

On the other hand, the abundance of small-molecular-weight polypeptides increased relative to the functional proteins in all samples dissolved in the presence of βME (Figure 2), which was expected due to the reduction in the disulfide linkages. However, dissolving the TGase-treated samples in SDS and in SDS and βME did not cause a significant increase in soluble aggregates nor functional proteins (Table 1), confirming that the polymers in the TG PPI and TG ChPI were formed by covalent linkages induced by transglutaminase. 

#### 2.1.3. Protein Denaturation

Two distinct, yet overlapping, endothermic peaks corresponding to vicilin and legumin were observed for the unmodified isolates (Table 2), in agreement with previous studies [12,14]. As for convicilin, its endothermic peak most likely overlapped with that of vicilin due to their similar structures [50]. Due to the overlapping endothermic peaks of vicilin and legumin, a combined enthalpy was calculated (Table 2).

The ChPI had a relatively larger legumin endothermic peak than that of vicilin (Figure S5), contrary to the PPI. This observation confirmed that the ChPI has a larger ratio of legumin to vicilin than that of the PPI (Figure 1). The higher abundance of legumin in the ChPI contributed to the significantly higher enthalpy than that of the PPI (Table 2), given the presence of disulfide linkages in legumin [22]. 

All commercial samples, except for the cChPC, did not show any endothermic peaks, indicating complete protein denaturation, attributed to the harsh wet extraction and processing conditions, as reported by Yaputri et al. [14]. Similarly, the TG PPI and TG ChPI did not show any endothermic peaks. During the TGase treatment, the PPI and ChPI were exposed to heat during enzyme incubation (50 °C) and enzyme inactivation (70 °C), which resulted in protein denaturation. In addition, it is possible that the structural changes induced by the TGase treatment further contributed to denaturation, not only to the tertiary structure, but also to the secondary structure of the protein.

#### 2.1.4. Protein Secondary Structure 

**Figure 3:**
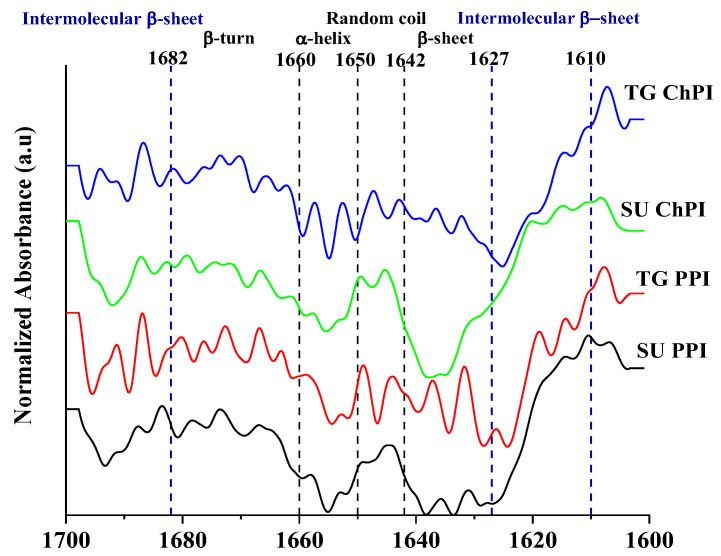
FTIR spectra of unmodified and TGase-modified pea and chickpea protein isolates (PPI, ChPI, TG PPI, and TG ChPI).

Wavenumbers 1610–1627 cm^−1^ and 1682–1700 cm^−1^ were assigned to the intermolecular β-sheet, while 1628–1642 cm^−1^ were assigned to the intramolecular β-sheet structures (Figure 3). On the other hand, wavenumbers 1643–1650 cm^−1^, 1650–1659 cm^−1^, and 1660–1681 cm^−1^ were assigned to the random coil, α-helix, and β-turn structures, respectively [51,52,53,54]. The secondary structures of the unmodified samples were mostly dominated by the β-sheet structures, followed by the α-helix or β-turn structures, and the random coil structures (Table 3). In comparison to the unmodified isolates, the TG PPI and TG ChPI had significantly higher intermolecular β-sheet, β-turn, and random coil structures, and significantly lower intramolecular β-sheet structures. These observations confirmed that the TG treatment resulted in the rearrangement of the secondary structures, including the unfolding and denaturation of the globular pea and chickpea proteins, and the subsequent formation of flexible structures like β-turns, as well as intermolecular β-sheet structures. The simultaneous effect of the TGase and heat during incubation and enzyme inactivation promoted protein denaturation and the consequent conversion of the α-helix and β-sheet into the intermolecular β-sheet, the predominant structures in aggregated proteins [49,53,55,56]. This indication of the presence of protein aggregates is complementary to the SE-HPLC and SDS-PAGE observations.

Inter- and intramolecular β-sheet structures have been associated with the enhanced gel strength of different proteins [24,56,57,58,59]. In addition, Zhang et al. [7] confirmed the importance of β-sheet structures to the formation of fibrous textures during the high-moisture extrusion of soy and gluten proteins for meat analog applications.

#### 2.1.5. Protein Surface Properties

Upon denaturation, globular proteins unfold and interior hydrophobic residues become exposed, resulting in an increase in the surface hydrophobicity [12,14,60]. Accordingly, the completely denatured TG ChPI had a significantly higher surface hydrophobicity compared to the unmodified ChPI (Table 2). However, the surface hydrophobicity of the TG ChPI was significantly lower compared to that of the cChPC, which was not completely denatured. In addition, the surface hydrophobicity of the TG PPI and that of the cPPI were not statistically different from that of the PPI. Both observations could be attributed to the protein polymerization caused by either the processing conditions or enzymatic crosslinking, which could have shielded some of the exposed hydrophobic sites of the denatured proteins due to steric hindrance, resulting in reduced surface hydrophobicity [12,60,61].

On the other hand, the TGase modification did not have any significant impact on the surface charge. In contrast, Glusac et al. [42] observed an increase in the net negative charge of TGase-modified chickpea protein. This discrepancy could be attributed to the deamidation of glutamine to glutamic acid by TG at a low substrate concentration (1%, *w*/*v*). In this study, the effect of deamidation on the surface charge was not observed at a higher protein concentration (5% *w*/*v*), since the deamidation rate is slower than the crosslinking of lysine and glutamine [62]. 

While there were some statistical differences in the surface charge among all tested samples, the values were not much different numerically (Table 2), except for that of the cSPI, which was evaluated as a reference. The slightly higher surface charge of the ChPI and TG ChPI compared to the PPI and TG PPI, respectively, could be attributed to the higher ratio of legumin to vicilin in the chickpea compared to pea protein. Legumin has a lower isoelectric point (a pH of 4.8) compared to vicilin (a pH of 5.5) [22], thus will have a higher net negative charge at a pH of 7. The ratio of the surface hydrophobicity to the surface charge, however, varied considerably across samples, most notably that of the TG ChPI compared to the ChPI, which may explain the functional behavior.

### 2.2. Impact of the TG-Induced Polymerization on the Protein Functional Properties

#### 2.2.1. Solubility

Protein solubility is an important functionality that is influenced by the surface properties, denaturation state, and the extent of the polymerization [12]. Therefore, the protein solubility of the TGase-modified isolates was evaluated against unmodified isolates and commercial reference ingredients at a pH of 7 under heated and nonheated conditions (Table 4). Heat treatment was included to mimic thermal food processing. 

Compared to the commercial samples, the PPI and ChPI had significantly higher solubility under all solubility conditions. The SE-UF process used to produce the PPI and ChPI was reported to preserve the protein’s structural integrity, resulting in superior solubility [12,14]. Similar to previous findings, the cPPI had the least solubility due to being completely denatured (Table 2) and highly polymerized (Figure 1). Such observations are attributed to the harsh wet extraction and processing conditions [12,14,23,63]. Although the cChPC was not completely denatured (Table 2), it had significantly higher surface hydrophobicity and a higher degree of denaturation compared to the ChPI. Such differences contributed to the significantly lower solubility of the cChPC compared to that of the ChPI (Table 4). In addition, the presence of a relatively high amount of starch in the cChPC could have also contributed to the reduced protein solubility, since the starch potentially competed with the protein for water. 

As expected, the TG PPI and TG ChPI had significantly lower solubility compared to the PPI and ChPI (Table 4). The decrease in the solubility of the TGase-modified isolates was attributed to their relatively high surface hydrophobicity, complete protein denaturation, protein polymerization, and significant increase in intermolecular β-sheets and random coils (Table 1, Table 2 and Table 3 and Figure 1, Figure 2 and Figure 3), in agreement with other reports on the TGase-modified pea and rice proteins [49,64]. However, the opposite solubility results were reported for the TGase-modified soy protein and pumpkin protein [65,66]. Such discrepancy could be attributed to different enzymatic treatment conditions, which could have led to varying surface properties and the extent of the protein crosslinking [62,67]. On the other hand, the heat treatment significantly increased the solubility of the commercial isolates and of the TG PPI and TG ChPI, an observation that could be attributed to the thermal kinetic energy that facilitates protein dispersibility [12,14]. The reduced solubility due to the TGase-induced denaturation and polymerization might prove beneficial for protein-network formation in thermally induced gels.

#### 2.2.2. Gel Strength

Gel strength is a measure of the protein network, which is influenced by the inherent protein profile, molecular size, surface properties, secondary structure, and the presence of sulfhydryl groups [22,50,68,69]. Protein–protein interactions via disulfide linkages, hydrogen bonding, and hydrophobic interactions have a positive impact on the gel formation and strength. Accordingly, the gel strength of the TG-modified isolates was evaluated against the unmodified isolates and commercial reference samples (Table 4). 

Compared to the unmodified isolates, the TG PPI and TG ChPI had a significantly superior gel strength (Table 4). Improvement in the gel strength following the TGase modification was also reported for the SPI and cPPI [70,71]. In the TGase-modified isolates, the relatively high-molecular-weight polymers (Figure 1) and lower % distribution of functional proteins (Figure 2 and Table 1) potentially contributed to the stronger protein-network formation [24]. In addition, the high relative abundance of intermolecular β-sheet and β-turn structures (Figure 3 and Table 3) contributed to the enhanced gel strength [24,56,57,58,59], mostly facilitated by hydrogen bonding [32,50,72]. The formation of hydrogen bonding increases the proximity of the denatured proteins, potentially resulting in disulfide interactions among the exposed sulfhydryl groups of the legumin subunits (Figure 1), contributing further to the gel strength. The TGase-induced polymerization in the SPI was accompanied by a decrease in the free sulfhydryl groups with the increasing TGase concentration in the SPI [70,73]. Another contributor to the enhanced protein–protein interactions is the relatively high surface hydrophobicity, which plays a significant role in bringing the protein polymers into close proximity. During the thermal treatment used to induce gel formation, intermolecular hydrophobic interactions are enhanced among unfolded proteins [50], contributing to the gel strength.

Of note is the significantly higher gel strength of the TG PPI and TG ChPI in comparison to their respective commercial counterparts. This observation indicated that the polymerization in the TG PPI compared to that of the cPPI is more ordered and in favor of forming a strong protein network. Proteins in the cPPI potentially are aggregated to an extent that could potentially disable the uniform gel-network formation [23], whereas the higher gel strength of the TG ChPI compared to the cChPC could mostly be attributed to the relatively higher abundance of high-molecular-weight polymers (Figure 1). 

The TG ChPI and ChPI had a significantly stronger gel strength than the TG PPI and PPI, respectively. In fact, the TG ChPI at a 20% protein concentration (*w*/*v*) had a comparable gel strength to the cSPI prepared at a 15% protein concentration (*w*/*v*) (Table 4). This observation could be attributed to the relatively higher ratio of legumin to vicilin in the chickpea protein compared to the pea protein (Figure 1). The presence of sulfhydryl groups in legumin promotes a strong protein network via disulfide interchange [50,74,75]. In addition, legumin monomers and subunits were more involved in TGase crosslinking compared to vicilin or smaller-MW proteins (Figure 1). However, genetic variance in legumin and vicilin among different species [22] could explain the need for the 20% protein concentration for the ChPI to form a cohesive gel compared to the 15% protein concentration needed for the SPI. Nevertheless, these findings confirmed the potential of TGase-induced crosslinking to enhance the protein network and gel strength of the pulse proteins.

#### 2.2.3. Emulsion Capacity

In general, good emulsion properties require proteins to have a good balance of surface hydrophilic and hydrophobic groups, a good solubility, and a flexible structure to interact with the water and oil phase [74,76,77]. Accordingly, the EC varied to some extent among the evaluated protein samples (Table 4).

The PPI and TG PPI had a significantly higher EC than the ChPI and TG ChPI, respectively. This observation can be attributed to a combination of factors, including the higher ratio of vicilin to legumin in the pea compared to chickpea protein (Figure 1) and the hydrophilic to hydrophobic balance on the surface of the protein (Table 2). The lower molecular weight of vicilin and its higher molecular flexibility compared to legumin contribute to the better emulsification properties [74,78]. The considerably lower surface hydrophobicity to charge ratio in the ChPI compared to the PPI could have caused the lower adsorption at the interface, resulting in the observed difference in the EC between the two samples. Further, the higher extent of the polymerization in the TG ChPI compared to the TG PPI (Figure 1, compare lane 7 to 4), discussed earlier, could partially explain the lower EC of the TG ChPI. Large polymers stabilized by covalent interactions of the legumin subunits (Figure 1) would have contributed to the reduced molecular flexibility and, consequently, the reduced EC. 

Both the PPI and TG PPI had a significantly higher EC than the cPPI. Again, this observation could be attributed to the excessively aggregated proteins in the cPPI. On the other hand, the EC of the cSPI was superior to all other samples, which could be attributed to the inherent differences in the protein fractions across species [22].

## 3. Conclusions

This study provided a comprehensive evaluation of the impact of TGase on the structure and function of PPI and ChPI produced following SE-UF at a pilot scale. Results demonstrated that the selected TGase treatment conditions successfully produced TG PPI and TG ChPI with high-molecular-weight polymers, and enhanced gel strength compared to the unmodified (PPI and ChPI) and commercial counterparts (cPPI and cChPC). Unique structural characteristics, including intermolecular β-sheet and β-turn structures, contributed to the enhanced gel strength, despite the significant reduction in the solubility. While the TGase modification did not enhance the emulsification, it did not have a detrimental effect. Results also revealed the potential impact of the legumin to vicilin ratio on the formation of covalently linked polymers, highlighting the inherent variation across species. Given the observed increase in the molecular weight and the resulting enhanced gel strength of the TG PPI and TG ChPI, such a modification could be leveraged for extruded meat analog products. Further investigation, therefore, is needed to evaluate the texturization potential of the TG PPI and TG ChPI as alternatives to soy protein in meat analogs. In addition, the scalability of such TGase modifications will need to be investigated. Nevertheless, this work provided a framework for a potentially feasible and successful approach to enhance the functionality of pulse proteins on a commercial scale.

## 4. Materials and Methods

### 4.1. Materials

Yellow field pea flour (21% protein), SYFP-100, was provided by AGT Foods (Regina, SK, Canada). Defatted chickpea flour (27% protein), Artesa™ Chickpea Flour 20M, and commercial chickpea protein concentration (cChPC) (56% protein, 5.3% ash), Artesa™ Chickpea Protein, were provided by Nutriati (Henrico, VA, USA). Commercial pea protein isolate (cPPI) (80% protein, 5.6% ash), ProFam^®^ 580, and commercial soy protein isolate (cSPI) (90% protein, 4.2% ash), ProFam^®^ 974, were provided by Archer Daniels Midland (ADM) (Decatur, IL, USA). Samples were stored at −20 °C until use. TGase (NSPP0037, 200 U/mL enzyme activity or 3.33 μkat/mL) was purchased from Novozymes (Bagsvaerd, Denmmark) and stored at 4 °C. Ultrafiltration membrane crossflow cassettes (3 kDa cut-off), Vivaflow^®^, were purchased from Sartorius™ (Gottingen, Germany). SnakeSkin™ dialysis tubing with a 3.5 kDa molecular weight cut-off (MWCO) was purchased from Thermo Fisher Scientific™ (Waltham, MA, USA). Criterion™ TGX 4–20% precast gels, 10X Tris/Glycine/sodium dodecyl sulfate (SDS) running buffer, Laemmli sample buffer, Precision Plus molecular weight marker, and Imperial™ Protein Strain were purchased from Bio-Rad Laboratories, Inc. (Hercules, CA, USA). Superdex™200 Increase 10/300 GL Prepacked Tricorn™ and gel filtration calibration kits for low molecular weights and HMWs were purchased from Cytiva (Marlborough, MA, USA). All other general lab supplies and analytical grade reagents were purchased from Thermo Fisher Scientific or Sigma-Aldrich (St. Louis, MO, USA).

### 4.2. Production of the PPI and ChPI 

The PPI and ChPI were produced on a pilot scale in the Joseph J. Warthensen Food Processing Center, University of Minnesota, following the SE-UF scaled-up process outlined by Hansen et al. [12] and Yaputri et al. [14] without modification. The protein content (PPI, 91%, and ChPI, 94%) was determined by the Dumas method (AOAC 990.03) using a LECO^®^ FP828 nitrogen analyzer (LECO, St. Joseph, MI, USA) with a conversion factor of 6.25. Ash content (PPI, 2.4%, and ChPI, 2.1%) was measured by the dry ashing method (AOAC 942.05). The produced isolates were stored at −20 °C until use.

### 4.3. Selection of the TGase Treatment Conditions and Production of the TG PPI and TG ChPI

Preliminary trials (varying enzyme concentrations and treatment times) at the optimal TGase enzyme temperature (50 °C) and pH (7.0) were performed. Controls without the addition of enzyme were performed simultaneously with each condition tested. TGase treatment conditions for each of the PPI and ChPI were selected (3.3 and 5 nkat/mL TGase, respectively, for 5 min) based on the observed protein polymerization (Figures S1–S4) and gel strength (Table S1) of the preliminary samples. The lengthy treatment time and relatively high enzyme concentration resulted in excessive polymerization and reduced gel strength (Figures S1–S4, Table S1). The TGase-modified PPI and ChPI (TG PPI and TG ChPI) were produced in triplicate under the selected conditions. Protein solutions (5% protein, *w*/*v*) were prepared in distilled, deionized water (DDW) and solubilized for 2 h at room temperature, followed by a pH adjustment to 7. Protein solutions were heated in a water bath and TGase was added once the temperature of the solution reached 50 °C. After incubation, the enzyme was immediately inactivated by reducing the pH to 3, followed by heating at 70 °C for 1 min. The pH was then adjusted back to 7, and all samples were dialyzed and lyophilized. The process was repeated until enough TGase isolates were obtained for characterization. Protein (TG PPI, 94%, and TG ChPI, 98%) and ash (TG PPI, 1.1%, and TG ChPI, 1.7%) contents were determined by Dumas and the dry ashing method, respectively.

### 4.4. Protein Structural Characterization 

#### 4.4.1. Protein Profiling by Gel Electrophoresis

Protein profiling by SDS polyacrylamide gel electrophoresis (SDS-PAGE) was performed as described by Hansen et al. [12]. Protein samples (4 mg protein/mL) were mixed 1:1 (*v*/*v*) with Laemmli buffer under nonreducing (without βME) and reducing (with βME) conditions. Aliquots (5 μL) of the protein samples (~0.05 mg protein) and Precision Plus™ MW standard were loaded onto 4–20% precast Tris-HCl gradient gels and electrophoresed at 200 V. Gels were stained, destained, and scanned using the Molecular Imager Gel Doc XR System (Bio-Rad Laboratories, Hercules, CA, USA).

#### 4.4.2. Protein Molecular Weight Distribution via Size-Exclusion High-Performance Liquid Chromatography (SE-HPLC)

The protein molecular weight distribution of all samples was analyzed using a Shimadzu HPLC system (Shimadzu Scientific Instruments, Colombia, MD, USA) and a Superdex 200 Increase 10/300 GL Prepacked Tricorn™ column following the SE-HPLC protocol reported by Bu et al. [23] without modification. In duplicate, 1% protein (*w*/*v*) samples were prepared in three different buffers: phosphate buffer with a pH of 7 (0.05 M sodium phosphate with 0.1 M NaCl), phosphate buffer with 0.1% SDS, and phosphate buffer with 0.1% SDS and 2.5% βME, and passed through a 0.45 μm filter before injection on the column to evaluate the degree of polymerization and protein association via noncovalent and covalent bonds. 

#### 4.4.3. Protein Denaturation

Protein denaturation temperature and enthalpy of all samples were evaluated in triplicate using a differential scanning colorimetry, DSC (Mettler Toledo, Columbus, OH, USA), as described by Bu et al. [23] without modification. Mettler Toledo’s STARe Software (version 11.00) was used to manually integrate the thermograms.

#### 4.4.4. Protein Secondary Structure by Attenuated Total Reflectance Fourier Transform Infrared Spectroscopy (ATR-FTIR)

ATR-FTIR spectra were obtained, in triplicate, for all samples in powder form. The spectra were recorded at the wavenumber range of 700 to 4000 cm^−1^ at room temperature using a Fourier transform infrared spectrometer (FTIR, Thermofisher Nicolett iS50) as outlined by Bu et al. [23]. OMNIC^®^ software (Thermo Scientific™ OMNIC™ Series Software, Waltham, MA, USA) was used for the data collection and integration. To obtain the best fit, a straight baseline passing through 1700–1600 cm^−1^ was adjusted. To determine the distribution of the protein secondary structures, the amide I region (1700–1600 cm^−1^) was deconvoluted. Baseline corrections, normalization, derivation, and curve fitting were performed using Origin 2018 software (OriginLab Corp., Northampton, MA, USA).

#### 4.4.5. Surface Properties of the Protein Ingredients

The protein surface charge of all samples was assessed in triplicate by measuring the zeta potential using a dynamic-light-scattering instrument (Malvern Nano Z-S Zetasizer) as outlined by Bu et al. [23] without modification. The zeta potential was determined using the Smoluchowski model with Malvern’s Zetasizer software (version 7.13). Protein surface hydrophobicity was also measured in triplicate using the spectrofluorometric method reported by Boyle et al. [79] and modified by Bu et al. [23]. 

### 4.5. Protein Functional Characterization 

#### 4.5.1. Protein Solubility

Protein solubility at a pH for all samples (5% protein solutions, *w*/*v*), with and without heating at 80 °C for 30 min, was determined in triplicate following the method reported by Boyle et al. [79]. The percent protein solubility was determined by measuring the protein content in the supernatant relative to the protein content in the original solution as analyzed using the Dumas method.

#### 4.5.2. Gel Strength

Thermally induced gels were prepared in triplicate, and their gel strength was measured as reported by Bu et al. [23], with modifications to the protein concentration and heating time. Thermally induced gels were formed at a 20% protein concentration (*w*/*v*) for the pea and chickpea proteins, and at a 15% protein concentration (*w*/*v*) for the cSPI. Protein samples were solubilized in DDW for 2 h and the pH was adjusted to 7. Sample aliquots (1 mL) were heated in a water bath at 95 °C (±2 °C) for 20 min (10 min for cSPI). After cooling, the gel strength was measured using a TA-XT Plus Texture Analyzer (Stable Micro Systems LTD, Surrey, UK) to record the maximum force (N) needed to rupture the gel.

#### 4.5.3. Emulsion Capacity

The emulsion capacity (EC) at 1% protein in DDW (*w*/*v*) of all the protein samples were determined in triplicate at a pH of 7, following a previously reported method [23] without modification. 

### 4.6. Statistical Analysis

IBM^®^ SPSS^®^ Statistics software version 26 for Windows (International Business Machines Corp., Armonk, NY, USA) was used for the one-way analysis of variance (ANOVA). The Tukey–Kramer multiple-means comparison test was used to determine the significant differences (*p* ≤ 0.05) among the means of three or more samples. For the means of two samples, a Student’s unpaired t-test was used to determine the significant differences (*p* ≤ 0.05).

## Figures and Tables

**Table 1:** Percent relative abundance of soluble aggregates, legumin, convicilin, and vicilin present in unmodified and TGase-modified pea and chickpea protein isolates (PPI, TG PPI, ChPI, and TG ChPI) as analyzed by size-exclusion high-performance chromatography (SE-HPLC).

Relative Abundance ^1^ (%) of Protein Fractions												
Samples	Phosphate Buffer ^2^				Phosphate Buffer (0.1% SDS) ^3^				Phosphate Buffer (0.1% SDS + 2.5% βME) ^4^			
	Soluble Aggregates (600–1200 kDa)	Legumin (~450 kDa)	Convicilin (~250 kDa)	Vicilin (~160 kDa)	Soluble Aggregates	Legumin	Convicilin	Vicilin	Soluble Aggregates	Legumin	Convicilin	Vicilin
PPI	5.73 ± 0.90 ^a,5^	21.2 ± 0.15 ^a^	6.16 ± 0.23 ^a^	5.01 ± 0.17 ^a^	14.4 ± 1.53 ^a^	19.5 ± 0.47 ^a^	5.54 ± 0.09 ^a^	4.64 ± 0.08 ^a^	13.4 ± 1.29 ^a^	13.0 ± 0.15 ^a^	3.92 ± 0.04 ^a^	3.17 ± 0.07 ^a^
TG PPI	1.77 ± 0.07 ^b^	5.83 ± 0.02 ^b^	3.84 ± 0.01 ^b^	2.03 ± 0.01 ^b^	6.21 ± 0.02 ^b^	4.05 ± 0.05 ^b^	3.81 ± 0.04 ^b^	2.19 ± 0.02 ^b^	4.71 ± 0.01 ^b^	2.00 ± 0.01 ^b^	2.19 ± 0.01 ^a^	1.34 ± 0.00 ^b^
ChPI	22.5 ± 1.67 ^A,6^	32.9 ± 0.78 ^A^	2.47 ± 0.08 ^B^	7.42 ± 0.17 ^A^	7.82 ± 0.16 ^A^	32.6 ± 0.56 ^A^	2.98 ± 0.01 ^B^	10.2 ± 0.12 ^A^	9.08 ± 0.24 ^A^	22.6 ± 0.03 ^A^	1.57 ± 0.01 ^A^	5.80 ± 0.01 ^A^
TG ChPI	10.2 ± 0.15 ^B^	6.70 ± 0.19 ^B^	5.25 ± 0.13 ^A^	6.55 ± 0.16 ^A^	7.62 ± 0.03 ^A^	5.91 ± 0.01 ^B^	4.85 ± 0.02 ^A^	7.33 ± 0.04 ^B^	1.80 ± 0.01 ^B^	2.65 ± 0.04 ^B^	1.65 ± 0.02 ^A^	2.30 ± 0.00 ^B^

^1^ Relative abundance (%) is the area of one peak relative to the total peak area; ^2^ Samples were dissolved in phosphate buffer at a pH of 7.0; ^3^ Samples were dissolved in phosphate buffer at a pH of 7.0 with 0.1% SDS; ^4^ Samples were dissolved in phosphate buffer at a pH of 7.0 with the addition of 0.1% SDS and 2.5% βME; ^5^ Lowercase letters denote significant differences between the means (*n* = 2, ±SE) of the TG PPI and PPI, and ^6^ uppercase letters denote significant differences between the means (*n* = 2) of the TG ChPI and ChPI, according to an independent sample *t*-test (*p* < 0.05).

**Table 2:** Denaturation temperature and enthalpy, surface hydrophobicity, and surface charge of unmodified and TGase-modified pea and chickpea protein isolates (PPI, TG PPI, ChPI, and TG ChPI), along with commercial samples (cSPI, cPPI, and cChPC).

Samples	Denaturation Temperature and Enthalpy			Surface Properties	
	Denaturation Temperature		Total Enthalpy of Denaturation (∆H)	Surface Hydrophobicity	Surface Charge
	°C		J g^−1^	RFI ^1^	mV
	β -conglycinin	Glycinin			
cSPI	* ^2^	*	*	10,800 ± 530.3 ^c^	−41.3 ± 0.20 ^a^
	Vicilin (7S)	Legumin (11S)			
cPPI	*	*	*	13,800 ± 434.4 ^a^	−30.2 ± 0.13 ^cd^
PPI	82.6 ± 0.13 ^a,3^	89.9 ± 0.16 ^c^	5.45 ± 0.07 ^b^	14,200 ± 105.9 ^a^	−27.2 ± 0.07 ^ef^
TG PPI	*	*	*	12,900 ± 203.6 ^ab^	−28.8 ± 0.28 ^de^
cChPC	81.5 ± 0.09 ^b^	99.6 ± 0.02 ^a^	3.77 ± 0.09 ^c^	13,300 ± 450.4 ^a^	−25.7 ± 0.33 ^f^
ChPI	80.5 ± 0.07 ^c^	90.8 ± 0.17 ^b^	8.61 ± 0.14 ^a^	8970 ± 186.5 ^d^	−30.9 ± 0.21 ^bc^
TG ChPI	*	*	*	11,600 ± 255.3 ^bc^	−32.2 ± 0.17 ^b^

^1^ Relative fluorescence index; ^2^ An asterisk (*) indicates the absence of peaks due complete protein denaturation; ^3^ Lowercase letters denote significant differences among the means (*n* = 3, ±SE) in each column according to the Tukey–Kramer multiple-means comparison test (*p* < 0.05).

**Table 3:** Protein secondary structure of unmodified and TGase-modified pea and chickpea protein isolates (PPI, TG PPI, ChPI, and TG ChPI).

Sample	Intermolecular β-Sheet	β-Sheet	α-Helix	β-Turn	Random Coil
PPI	39.8 ± 0.39 *	32.1 ± 0.53 *	18.7 ± 0.28 *	8.10 ± 0.72 *	2.13 ± 0.50 *
TG PPI	58.1 ± 2.26	11.7 ± 2.08	13.3 ± 0.34	12.1 ± 0.50	4.71 ± 0.02
ChPI	17.5 ± 1.04 *	55.7 ± 1.01 *	17.9 ± 1.29	7.81 ± 0.40 *	1.08 ± 0.11 *
TG ChPI	47.8 ± 0.54	11.6 ± 0.09	16.1 ± 0.29	17.9 ± 0.56	6.59 ± 0.68

* Indicates significant difference between the means (*n* = 2, ±SE) of each sample type (i.e., PPI and TG PPI; ChPI and TG-ChPI) as tested by the independent samples t-test (*p <* 0.05).

**Table 4:** Protein solubility, gel strength, and emulsion capacity of unmodified and TGase-modified pea and chickpea protein isolates (PPI, ChPI, TG PPI, and TG ChPI), along with commercial samples (cSPI, cPPI, and cChPC).

Samples	% Protein Solubility		Gel Strength ^1^ (N)	Emulsion Capacity (mL oil/g Protein)
	Nonheated	Heated ^2^		
cSPI	66.8 ± 0.40 ^c,3^	78.5 ± 0.39 ^b,^*^,4^	19.2 ± 0.09 ^a^	1120 ± 10.7 ^a^
cPPI	29.5 ± 0.85 ^d^	57.1 ± 0.64 ^e,*^	11.5 ± 0.16 ^d^	769 ± 6.20 ^d^
PPI	68.9 ± 0.42 ^bc^	69.9 ± 0.90 ^c^	10.7 ± 0.11 ^d^	856 ± 10.7 ^b^
TG PPI	20.8 ± 1.07 ^e^	25.6 ± 0.44 ^g,^*	16.2 ± 0.26 ^b^	843 ± 6.20 ^bc^
cChPC	70.7 ± 0.31 ^b^	66.7 ± 0.47 ^d,^*	13.3 ± 0.33 ^c^	750 ± 16.4 ^d^
ChPI	94.3 ± 0.69 ^a^	92.5 ± 0.76 ^a^	15.3 ± 0.17 ^b^	794 ± 12.4 ^cd^
TG ChPI	23.4 ± 1.5 ^e^	32.6 ± 0.39 ^f,^*	18.4 ± 0.42 ^a^	688 ± 11 ^e^

^1^ The cSPI gel strength was determined at 15% protein (*w*/*v*), while all other samples were determined at 20% protein (*w*/*v*); ^2^ Heated at 80 °C for 30 min; ^3^ Lowercase letters denote significant differences among the means (*n* = 3, ±SE) in each column according to the Tukey–Kramer multiple-means comparison test (*p* < 0.05); ^4^ An asterisk (*) denotes significant differences between nonheated and heated samples according to the Student’s unpaired *t*-test (*p* < 0.05).

## Data Availability

Raw data collected are available upon request from the corresponding author. Raw data was used to generate the summary of data in the presented tables and figures.

## Supplementary Materials

The following supporting information can be downloaded at: https://www.mdpi.com/article/10.3390/gels10010011/s1, Figure S1: SDS-PAGE gel protein profile visualization of PPI upon TGase treatment (C = 0 nkat/mL enzyme and E = 67 nkat/mL enzyme) for different times under nonreducing and reducing conditions. Lane 1: molecular weight (MW) marker; lanes 2, 15: PPI; lanes 3–4, 16–17: C and E 5 min; lanes 5–6, 18–19: C and E 10 min; lanes 7–8, 20–21: C and E 15 min; lanes 9–10, 22–23: C and E 30 min; lanes 11–12, 24–25: C and E 45 min; lanes 13–14, 26–27: C and E 60 min. Lox: lipoxygenase; Cs: convicilin subunits; Lm: legumin monomer; Vs: vicilin subunits; Lsα: legumin acidic subunits, Lsβ: legumin basic subunits; Vsf: vicilin subunit fractions due to post-translational cleavages; Figure S2: SDS-PAGE gel protein profile visualization of PPI treated with TGase for 5 min at different enzyme concentrations under nonreducing (lanes 2–9) and reducing conditions (lanes 10–17). Lane 1: molecular weight (MW) marker; lanes 2, 10: PPI; lanes 3, 11: 0 nkat/mL enzyme; lanes 4, 12: 0.8 nkat/mL enzyme; lanes 5, 13: 1.7 nkat/mL enzyme; lanes 6, 14: 3.3 nkat/mL enzyme; lanes 7, 15: 8.3 nkat/mL enzyme; lanes 8, 16: 17 nkat/mL enzyme; lanes 9, 17: 33 nkat/mL enzyme. Lox: lipoxygenase; Cs: convicilin subunits; Lm: legumin monomer; Vs: vicilin subunits; Lsα: legumin acidic subunits, Lsβ: legumin basic subunits; Vsf: vicilin subunit fractions due to post-translational cleavages; Figure S3: SDS-PAGE gel protein profile visualization of ChPI upon TGase treatment (C = 0 nkat/mL enzyme and E = 67 nkat/mL enzyme) for different times under nonreducing (lanes 2–9) and reducing conditions (lanes 10–17). The enzyme activity of the enzyme stock is 3.3 μkat/mL. Lane 1: molecular weight (MW) marker; lanes 2, 10: 15 min C; lanes 3, 11: 15 min E; lanes 4, 12: 30 min C; lanes 5, 13: 30 min E; lanes 6, 14: 45 min C; lanes 7, 15: 45 min E; lanes 8, 16: 60 min C; lanes 9, 17: 60 min E. Lox: lipoxygenase; C_s_: convicilin subunits; L_m_: legumin monomer; V_s_: vicilin subunits; L_s_α: legumin acidic subunits, L_s_β: legumin basic subunits; V_s_f: vicilin subunit fractions due to post-translational cleavages; Figure S4: SDS-PAGE gel protein profile visualization of ChPI treated with TGase for 5 min at different enzyme concentrations under nonreducing (lane 2–9) and reducing conditions (lane 10–17). The enzyme activity of the enzyme stock is 3.3 μkat/mL. Lane 1: molecular weight (MW) marker; lanes 2, 10: SU ChPI; lanes 3, 11: 0 nkat/mL enzyme; lanes 4, 12: 0.8 nkat/mL enzyme; lanes 5, 13: 1.7 nkat/mL enzyme; lanes 6, 14: 3.3 nkat/mL enzyme; lanes 7, 15: 5 nkat/mL enzyme; lanes 8, 16: 6.7 nkat/mL enzyme; lanes 9, 17: 8.3 nkat/mL enzyme. Lox: lipoxygenase; Cs: convicilin subunits; Lm: legumin monomer; Vs: vicilin subunits; Lsα: legumin acidic subunits, Lsβ: legumin basic subunits; Vsf: vicilin subunit fractions due to post-translational cleavages; Figure S5: DSC graph of unmodified and TG-modified pea and chickpea protein isolates (PPI, ChPI, TG PPI, and TG ChPI); Table S1: Gel strength of PPI and ChPI treated with different TG concentrations for different incubation times.
