# Supplementary material for: Transglutaminase-Induced Polymerization of Pea and Chickpea Protein to Enhance Functionality
Source: Gels. 2023 Dec 22;10(1):11. doi: 10.3390/gels10010011 (PMC10815621; doi:10.3390/gels10010011)
Supplement: Supplementary file 1 [file gels-10-00011-s001.zip › gels-2721742-supplementary.pdf]

Supplementary Material

Transglutaminase-Induced Polymerization of Pea and Chickpea Protein to Enhance Functionality

Brigitta P Yaputri[a] ,Samira Feyzi[a] and Baraem P Ismail[a*]

[a]Food Science and Nutrition Department, University of Minnesota, 1334 Eckles Ave, Saint Paul, Minnesota 55108, USA

*Correspondence: Email: bismailm@umn.edu

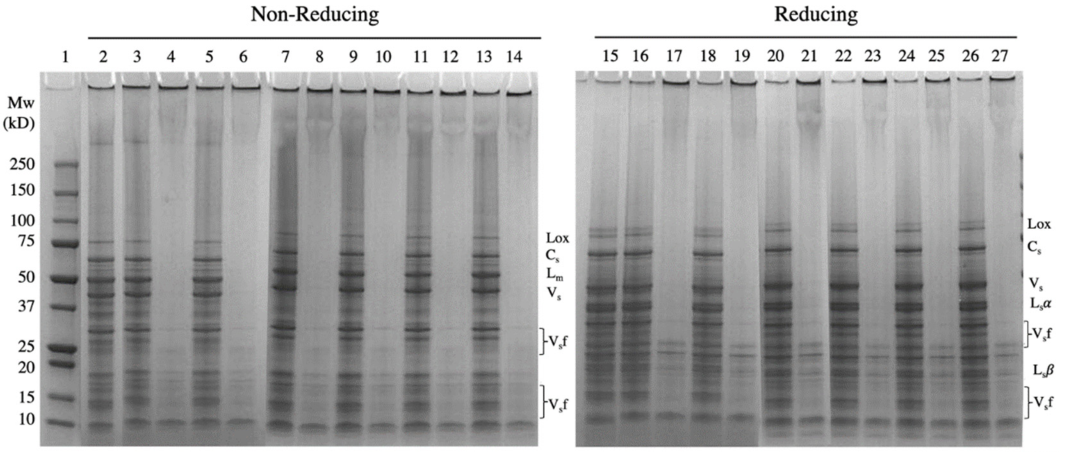

Figure S1. SDS-PAGE gel protein profile visualization of PPI upon TG treatment (C = 0 nkat/mL enzyme and E = 67 nkat/mL enzyme) for different times under non-reducing and reducing conditions. Lane 1: Molecular weight (MW) marker; lanes 2,15: PPI; lanes 3-4, 16-17: C and E 5 min; lanes 5-6, 18-19: C and E 10 min, lanes 7-8, 20-21: C and E 15 min; lanes 9-10, 22-23: C and E 30 min; lanes 11-12, 24-25: C and E 45 min; lanes 13-14, 26-27: C and E 60 min. Lox: lipoxygenase; C_s: convicilin subunits; L_m: legumin monomer; V_s: vicilin subunits; $L_s\alpha$: legumin acidic subunits, $L_s\beta$: legumin basic subunits; V_sf: vicilin subunit fractions due to post-translational cleavages.

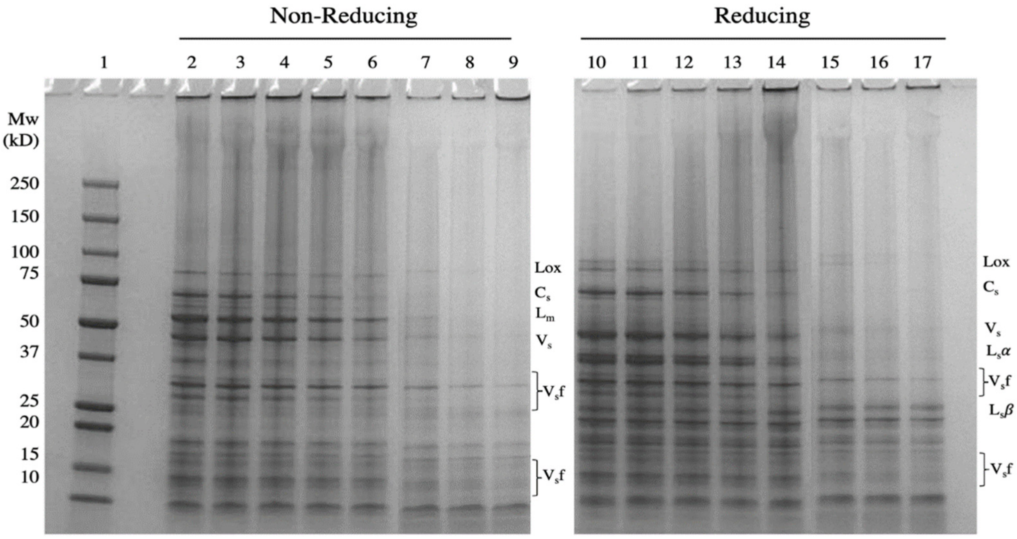

Figure S2. SDS-PAGE gel protein profile visualization of PPI treated with TG for 5 minutes at different enzyme concentrations under non-reducing (lanes 2-9) and reducing conditions (lanes 10-17). Lane 1: Molecular weight (MW) marker; lanes 2,10: PPI; lanes 3,11: 0 nkat/mL enzyme; lanes 4,12: 0.8 nkat/mL enzyme; lanes 5,13: 1.7 nkat/mL enzyme; lanes 6,14: 3.3 nkat/mL enzyme; lanes 7,15: 8.3

nkat/mL enzyme; lanes 8,16: 17 nkat/mL enzyme; lanes 9,17: 33 nkat/mL enzyme. Lox: lipoxygenase; C_s: convicilin subunits; L_m: legumin monomer; V_s: vicilin subunits; $L_s\alpha$: legumin acidic subunits, $L_s\beta$: legumin basic subunits; V_sf: vicilin subunit fractions due to post-translational cleavages.

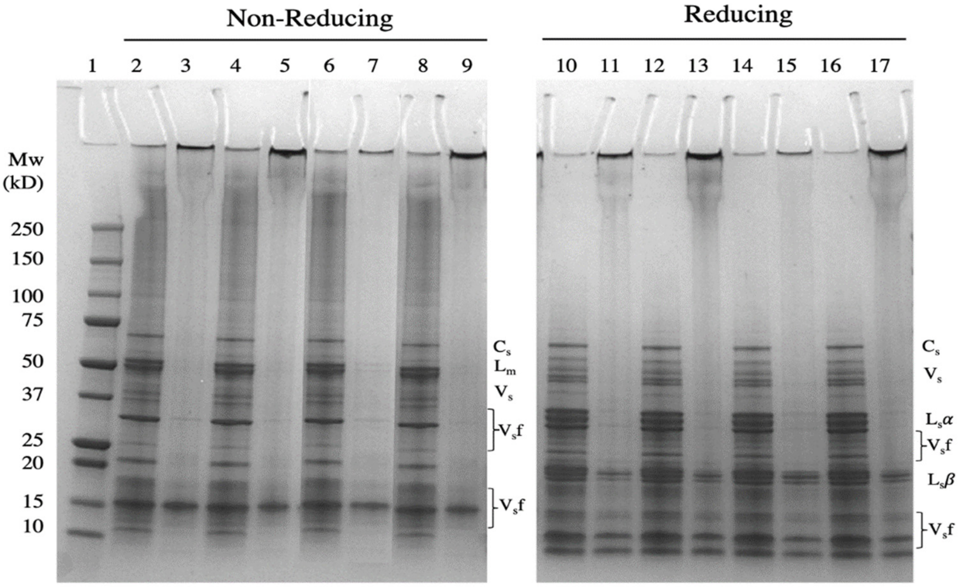

Figure S3. SDS-PAGE gel protein profile visualization of ChPI upon TG treatment (C = 0 nkat/mL enzyme and E = 67 nkat/mL enzyme) for different times under non-reducing (lanes 2-9) and reducing conditions (lanes 10-17). The enzyme activity of the enzyme stock is 3.3 μkat/mL. Lane 1: Molecular weight (MW) marker; lanes 2,10: 15 min C; lanes 3,11: 15 min E; lanes 4,12: 30 min C; lanes 5,13: 30 min E; lanes 6,14: 45 min C; lanes 7,15: 45 min E; lanes 8,16: 60 min C; lanes 9,17: 60 min E. Lox: lipoxygenase; C_s: convicilin subunits; L_m: legumin monomer; V_s: vicilin subunits; $L_s\alpha$: legumin acidic subunits, $L_s\beta$: legumin basic subunits; V_sf: vicilin subunit fractions due to post-translational cleavages.

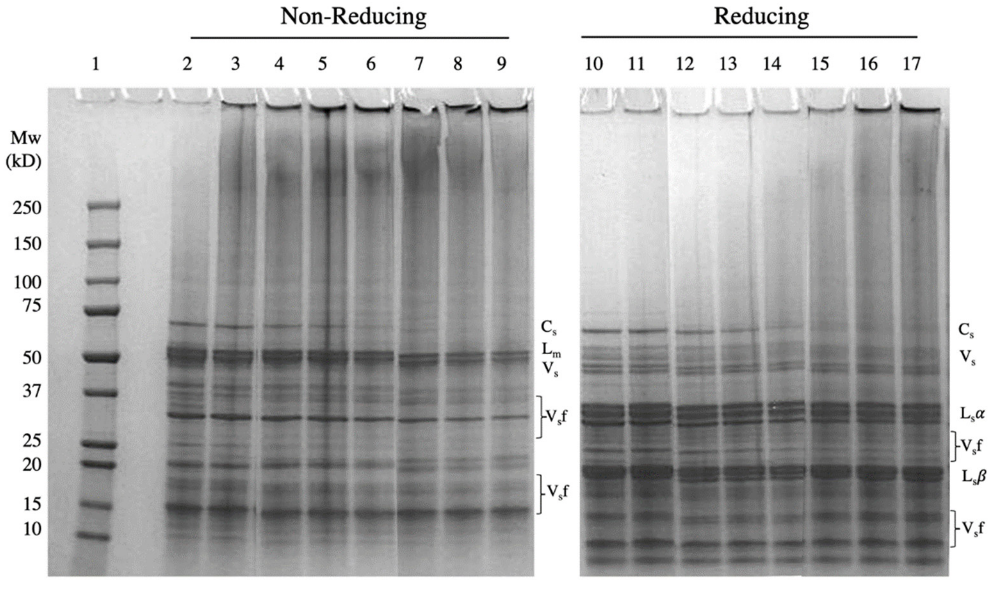

Figure S4. SDS-PAGE gel protein profile visualization of ChPI treated with TG for 5 minutes at different enzyme concentrations under non-reducing (lane 2-9) and reducing conditions (lane 10-17). The enzyme activity of the enzyme stock is 3.3 μkat/mL. Lane 1: Molecular weight (MW) marker; Lanes 2,10: SU ChPI; Lanes 3,11: 0 nkat/mL enzyme; Lanes 4,12: 0.8 nkat/mL enzyme; Lanes 5,13: 1.7 nkat/mL enzyme; Lanes 6,14: 3.3 nkat/mL enzyme; Lanes 7,15: 5 nkat/mL enzyme; Lanes 8,16: 6.7 nkat/mL enzyme; Lanes 9,17: 8.3 nkat/mL enzyme. Lox: lipoxygenase; C_s: convicilin subunits; L_m: legumin monomer; V_s: vicilin subunits; $L_s\alpha$: legumin acidic subunits, $L_s\beta$: legumin basic subunits; V_sf: vicilin subunit fractions due to post-translational cleavages.

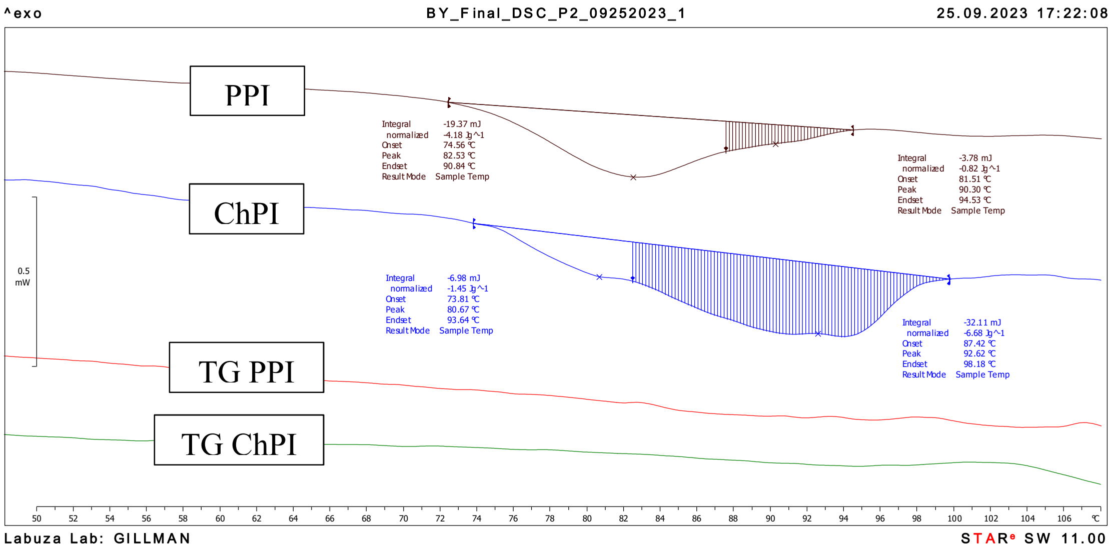

Figure S5. DSC graph of unmodified and TG-modified pea and chickpea protein isolates (PPI, ChPI, TG PPI, and TG ChPI).

Table S1. Gel strength of PPI and ChPI treated with different TG concentration for different incubation times.

Sample	Protein % (w/v)	Enzyme activity (nkat/mL)[1]	Treatment time	Gel strength (N)
TG PPI	20%	0	0	13.2 ± 0.16^{bc}
		67	60	6.76 ± 0.17^{g}
		0		10.3 ± 0.20^{ef}
		67	45	6.80 ± 0.21^{g}
		0		11.9 ± 0.09^{de}
		67	30	10.5 ± 0.51^{ef}
		0		13.4 ± 0.18^{bc}
		67	15	9.16 ± 0.05^{f}
		0		11.1 ± 0.12^{de}
		3.3	5	16.5 ± 0.59^{a}
		1.7		13.9 ± 0.26^{b}
		0.8		12.5 ± 0.49^{cd}
		0		9.81 ± 0.09^{f}
TG ChPI	20%	0	0	19.1 ± 0.50^{B}
		67	60	23.0 ± 0.73^{A}
		0		11.8 ± 0.16^{CD}
		67	45	9.41 ± 0.31^{E}
		0		10.9 ± 0.25^{DE}
		67	30	19.8 ± 0.62^{B}
		0		12.0 ± 0.32^{C}
		67	15	5.17 ± 0.87^{F}
		0		10.6 ± 0.21^{DE}
	15%	0	5	$3.88 \pm 0.03^{\beta}$
		8.3		$4.97 \pm 0.10^{\alpha\beta}$
		6.7		$5.83 \pm 0.32^{\alpha\beta}$
		5.0		$6.78 \pm 0.28^{\alpha}$
		3.3		$6.33 \pm 0.35^{\alpha}$
		1.7		$5.96 \pm 0.21^{\alpha}$
		0.8		$5.44 \pm 0.20^{\alpha\beta}$

[1]nkat/mL refers to enzyme activity per sample volume; [2]Lowercase letters denote significant differences among the means (n=3, ± SE) of TG PPI gel strength measured at 20% (w/v)); [3]Uppercase letters denote significant differences among the means (n=3, ± SE) of TG ChPI gel strength measured at 20% (w/v); [3]Greek letters denote significant differences

among the means (n=3, ± SE) of TG ChPI gel strength measured at 15% (w/v) according to the Tukey-Kramer multiple means comparison test ($P < 0.05$).
